# Cellular Antioxidant Effect of an Aronia Extract and Its Polyphenolic Fractions Enriched in Proanthocyanidins, Phenolic Acids, and Anthocyanins

**DOI:** 10.3390/antiox11081561

**Published:** 2022-08-12

**Authors:** Cécile Dufour, Jose A. Villa-Rodriguez, Christophe Furger, Jacob Lessard-Lord, Camille Gironde, Mylène Rigal, Ashraf Badr, Yves Desjardins, Denis Guyonnet

**Affiliations:** 1 Anti Oxidant Power AOP/MH2F-LAAS/CNRS, 7 Avenue du Colonel Roche, BP 54200, 31031 Toulouse, France; 2 Business Incubation Group, Symrise Taste, Nutrition & Health, 1E Allée Ermengarde d’Anjou, 35000 Rennes, France; 3 Institute of Nutrition and Functional Foods (INAF), Université Laval, Quebec, QC G1V 0A6, Canada

**Keywords:** aronia, chokeberry, (poly)phenols, proanthocyanidins, phenolic acids, anthocyanins, oxidative stress, NRF2, antioxidant cell bioassays

## Abstract

Oxidative stress and chronic inflammation contribute to some chronic diseases. Aronia berries are rich in polyphenols. The aim of the present study was to characterize the cellular antioxidant effect of an aronia extract to reflect the potential physiological in vivo effect. Cellular in vitro assays in three cell lines (Caco-2, HepG2, and SH-SY5Y) were used to measure the antioxidant effect of AE, in three enriched polyphenolic fractions (A1: anthocyanins and phenolic acids; A2: oligomeric proanthocyanidins; A3: polymeric proanthocyanidins), pure polyphenols and microbial metabolites. Both direct (intracellular and membrane radical scavenging, catalase-like effect) and indirect (NRF2/ARE) antioxidant effects were assessed. AE exerted an intracellular free radical scavenging activity in the three cell lines, and A2 and A3 fractions showed a higher effect in HepG2 and Caco-2 cells. AE also exhibited a catalase-like activity, with the A3 fraction having a significant higher activity. Only A1 fraction activated the NRF2/ARE pathway. Quercetin and caffeic acid are the most potent antioxidant polyphenols, whereas cyanidin and 5-(3′,4′-dihydroxyphenyl)-γ-valerolactone showed the highest antioxidant effect among polyphenol metabolites. AE rich in polyphenols possesses broad cellular antioxidant effects, and proanthocyanidins are major contributors. Polyphenol metabolites may contribute to the overall antioxidant effect of such extract in vivo.

## 1. Introduction

A body of evidence suggest that the intake of plant-based foods rich in fibers, vitamins, minerals, and phytochemicals is beneficial for long-term health. A meta-analysis of eight cohort studies concluded that higher adherence to the Mediterranean diet, which is characterized by a high intake of cereals, vegetables, fruits, nuts, and olive oil, significantly reduces the overall mortality, mortality from cardiovascular diseases, cancer, and incidence of Parkinson’s and Alzheimer’s disease [1]. Similarly, epidemiological and dietary intervention studies indicate that the regular consumption of fruits and vegetables lowers the risks of developing cancers, cardiovascular diseases, or obesity [2,3]. An integral component of plant-based items is the content in micronutrients and phytochemicals able to regulate redox status in the human body, therefore helping to counteract oxidative stress.

Elevated oxidative stress and chronic inflammation are believed to contribute to the pathophysiology of chronic diseases such as diabetes, cancer, cardiovascular disease, neurodegenerative diseases, and aging [4,5]. In this regard, berries have been the subject of a number of health-related studies because they stand out for their levels of antioxidant components including carotenoids and vitamin C [6]; however, their high polyphenol content has generated the most attention.

One berry of increasing interest is *Aronia melanocarpa* (Michx.) Elliot (chokeberry) which displays amongst the highest polyphenol content when compared with other berries such as blueberries, blackberries, blackcurrant, grapes, lingonberries, cranberries, raspberries, and strawberries [7,8], and has a higher antioxidative potential based on in vitro radical-scavenging assays such as DPPH, FRAP, or ORAC [8,9,10]. Aronia is a prominent source of anthocyanins, proanthocyanidins (PACs), flavonols (such as quercetin and its glycosides), and hydroxycinnamic acids (particularly caffeoylquinic acid) [10,11,12,13]. They are notably rich in PAC polymers with a high degree of polymerization. This large spectrum of polyphenolic compounds and their content makes aronia an interesting target for the development of functional foods, dietary supplements, and even therapeutics to deliver these molecules in a calibrated manner to protect and reduce the risk and onset of non-communicable conditions linked to oxidative stress and associated inflammation (e.g., diabetes, cardiovascular disease, and neurodegeneration). Indeed, polyphenols present in aronia have been shown to exert antioxidant, anti-inflammatory, or immunomodulatory effects, thus helping to protect cellular integrity and organ function.

Anthocyanins are a class of polyphenols that have been extensively characterized for their antioxidant activity and potential to modulate oxidative stress and related metabolic disorders including diabetes, cardiovascular diseases, and cognitive decline [7,14,15,16,17]. As a result, pre-clinical [18,19] and human intervention studies [20,21] addressing the protective effects of aronia have largely attributed the effects observed to the anthocyanin content, and the contribution of PACs has not been taken into consideration, which may be partially due to their high structural complexity. Importantly, some reports suggest that PACs are found in aronia fruit and juice at a higher concentration than anthocyanins [11,22], and these compounds virtually mirror all the protective effects described for anthocyanins, including strong antioxidant properties and cardiovascular and neurocognitive benefits [23,24,25,26]. Notably, most of the ingested PACs are not absorbed in the small intestine and reach the colon, where they can modify the redox status of the colonic environment and re-shape resident microbial communities [27,28]. Furthermore, resident bacteria in the colon transform PACs into smaller bioavailable catabolites, which may not only influence local, but also distant redox environment in tissues such as the brain and liver, something that remains largely understudied so far. Indeed, microbiota-generated catabolites of PACs such as phenyl-γ-valerolactones have shown some potential health effects in pre-clinical models of colorectal cancer and neurodegeneration [29,30].

Based on the above, the aim of present study was to characterize the cellular antioxidant effect of: (i) an aronia extract (AE) rich in polyphenols; (ii) PAC oligomers and polymers in AE versus other major polyphenols; and (iii) common metabolites produced after the intake of anthocyanins, hydroxycinnamic acids, PACs, and quercetin glycosides. Unlike many other studies addressing the antioxidant activity of polyphenols by chemical “cell-free” assays, the characterization of the antioxidant effects conducted in this study was performed using cellular in vitro assays, which more accurately reflect the potential efficacy in an in vivo physiological context.

## 2. Materials and Methods

### 2.1. Chemicals for Cell Biology

Quercetin, epicatechin, chlorogenic acid (>95%), trans-ferulic acid (99%), caffeic acid (>98%), vanillic acid (>97%), hippuric acid (98%), cyanidin chloride, cyanidin 3-O-galactoside chloride, sulforaphane, thiazole orange (TO), 2,2-Azobis(2-methylpropionamidine) dihydrochloride (AAPH), and 2′,7′-Dichlorofluorescin diacetate (DCFH-DA) were purchased from Sigma-Aldrich (Saint-Quentin Fallavier, France). Procyanidin B2 was purchased from Extrasynthèse (Genay, France) and (4R) 5-(3′,4′-dihydroxyphenyl)-γ-valerolactone (3,4-DHPVL) (90% enantiomeric excess) was purchased from Enamine (Riga, Latvia). Gibco DMEM (high-glucose, GlutaMAX supplement and pyruvate), fetal bovine serum (FBS) (HyClone, Logan, Utah, United States), pen-strep solution (100X) (Gibco), 0.05% Trypsin-EDTA (HyClone), Gibco Selective Antibiotic Geneticin (G418) (50 mg/mL), Gibco DPBS without Calcium and Magnesium (1X) were purchased from Thermo Fisher Scientific (Illkirch-Graffenstaden, France). HepG2 (catalogue number HB8065) and SH-SY5Y (catalogue number CRL-2266) cell lines were purchased from the American Type Cell Collection (ATCC) (LGC Standards, Molsheim, France). The Caco-2 cell line was a gift from Led Engineering Development (LED, Montauban, France). ARE Reporter—HepG2 Cell Line (catalogue number 60513) were purchased from BPS Bioscience (San Diego, CA, USA).

### 2.2. Aronia Extract and Fractions

*Aronia melanocarpa* (Michx.) Elliot extract and its polyphenolic fractions were provided by Symrise TN&H (Québec, Canada). AE powder was obtained by a purification process from Aronia juice concentrate as follows: Aronia juice concentrate was diluted with water. The diluted concentrate was then loaded onto a column containing Amberlite XAD-7HP absorbent resin. After loading, an acidified water wash was used to remove sugars and organic acids from the resin column. The phenolic components were then eluted from the resin column using an ethanol water solvent mixture. The phenolic fraction was partially concentrated, and the ethanol was recovered, before the concentrated extract was vacuum dried to yield the final powder.

AE was fractionated by gel-filtration chromatography, as described by Rodriguez-Daza et al. [31] to produce three different aronia fractions enriched in different polyphenols: fraction A1, rich in anthocyanins and phenolic acids; fraction A2, rich in PAC oligomers; fraction A3, rich in PAC polymers (A3). PAC oligomers were defined as PAC with a degree of polymerization (DP) ≤4 and PAC polymers as PAC with a DP > 4 [31]. Briefly, 75 g/L of crude AE solubilized in acidified water (1% acetic acid) was deposited on 30 g of cation exchange resin (siliaPrepX SCX, Silicycle, Canada), and the AE was eluted with 50% ethanol (polyphenols containing fraction). This fraction was placed on a Sephadex LH-20 column (30 × 600 mm) that had previously been hydrated for more than 2 h in 20% aqueous methanol. The column was eluted sequentially with 500 mL of 60% methanol acidified with 5% of HCl to obtain anthocyanins and phenolic acids fraction (A1); eluted with 1 L of methanol to obtain the PAC oligomers fraction (A2); and then with 2 L of 70% acetone/water (*v*/*v*) to obtain the PAC polymers fraction (A3). The respective fractions were then evaporated to remove solvents and lyophilized to remove residual water. For the cell-based assays, the AE and fractions were solubilized at a final concentration of 50 mg/mL in cell culture medium. Solutions were centrifuged at 8700 rpm for 10 min and supernatants were collected, aliquoted, and stored at −20 °C before the preparation of each range of serial dilutions.

### 2.3. Characterization of the Aronia Extract and Fractions

#### 2.3.1. Analysis of Anthocyanins by HPLC-Vis

Quantification of anthocyanins was performed as previously described, with slight modifications [32]. The column used was a Zorbax Stablebond SB-C18 (4.6 × 250 mm, 5 μm) (Agilent Technologies, Santa Clara, CA, USA) and the gradient was as follows: 0–2 min: 5% B, 2–10 min: 5–20% B, 10–15 min: 20% B, 15–30 min: 20–25% B, 30–35 min: 25% B, 35–50 min: 25–33% B, 50–55 min: 33% B, 55–65 min: 33–36% B, 65–70 min: 36–45% B, 70–75 min: 45–53% B, 75–80 min: 53–55% B, 80–84 min: 55–70% B, 84–85 min: 70–5% B and 85–90 min: 5% B. Cyanidin 3-glucoside was used for quantification. Each sample was injected in triplicate.

#### 2.3.2. Analysis of Phenolic Acids, Flavan-3-Ols, PAC and Flavonols by UPLC-UV-QToF

The method was adapted from Jakobek et al. [33]: 25 mg of sample was dissolved in 1 mL of methanol:water (80:20 *v*/*v*) acidified with 1% of formic acid. Samples were vortexed, sonicated for 15 min, and filtered through a 0.22 µm Nylon filter (Chromatographic Specialities Inc., Brockville, ON, Canada). Samples were further diluted in methanol:water (80:20 *v*/*v*) acidified with 1% of formic acid when necessary for adequate quantification. Stock solutions of epicatechin, procyanidin B2, chlorogenic acid, and quercetin were prepared in methanol at a concentration of 10,000 ppm. Calibration curves were prepared by diluting the stock solution in methanol:water (80:20 *v*/*v*) acidified with 1% of formic acid at different concentrations (1, 2, 4, 6, 8, 10, 25, 50, 100, 150, 200, 250, and 500 ppm).

Analysis was carried out with an ACQUITY I-Class UPLC-UV coupled with a Synapt G2-Si QtoF (Waters, Milford, MA, USA): 1 µL was injected onto an ACQUITY Premier HSS T3 column (2.1 × 100 mm, 1.8 μm) (Waters, Milford, MA, USA) with an ACQUITY Premier HSS T3 VanGuard FIT cartridge (2.1 × 5 mm, 1.8 μm) (Waters, Milford, MA, USA) heated to 40 °C. Mobile phase A (water) and mobile phase B (acetonitrile) were acidified with 1% of formic acid. The gradient was as follows, with a flow rate of 0.4 mL/min: 0–1 min: 3% B, 1–15 min: 3–13.5% B, 19–25 min: 18.5–30% B, 25–27.9 min: 30–37.3% B, 27.9–28 min: 37.3–95% B, 28–30.9 min: 95% B, 30.9–31 min: 95–3% B and–34 min: 3% B. UV signals were acquired at 280 nm (flavan-3-ols and PAC), 320 nm (phenolic acids) and 360 nm (flavonols) with a resolution of 2.4 nm and a sampling rate of 20 Hz. Samples were kept at 4 °C in the autosampler compartment. The source parameters were as follows: capillary voltage, −2.40 kV; source temperature, 150 °C; desolvation temperature, 600 °C; cone gas flow, 50 L/h and desolvation gas, 1000 L/h. Leucine–enkephaline (200 pg/μL) was infused at a flow rate of 10 μL/min for use as internal mass standard.

The extract and fractions were injected in full MS in negative electrospray ionization and resolution mode (resolution ≈ 25,000) with a scan range (m/z) of 50 to 2000 and a scan time of 0.2 s to determine the mass of each peak present in the UV chromatograms (280, 320 and 360 nm). Then, the samples were re-injected in Fast-DDA mode with an inclusion list to fragment each phenolic compound detected in the first injection. Data were acquired in negative electrospray ionization and resolution mode. The MS survey scan parameters was the same as the full MS acquisition. For each MS Survey scan, three MS/MS scans were performed with a scan time of 0.1 s and a collision energy ranging from 15 to 45 V. Only ions in the inclusion list within +/− 20 mDa and +/− 20 s were fragmented. Compound identification was performed using Progenesis QI v2.4 software. When an authentic standard was not available, identification was performed using Phenol-Explorer as a database. The search was conducted with a precursor tolerance of 10 ppm and was filtered by an isotope similarity score higher than 90%. Theoretical fragmentation was performed with a fragment tolerance of 10 ppm. After identification, phenolic compounds were quantified using UV data with TargetLynx XS v4.2 software (Waters, Milford, MA, USA). Flavan-3-ols were quantified as epicatechin equivalent, phenolic acids as chlorogenic equivalent, and flavonols as quercetin equivalent. Each sample were injected in triplicate.

#### 2.3.3. Determination of Total PAC Content and Mean DP Using Phloroglucinolysis by UPLC-UV-QtoF

The method was adapted from [33]. In brief, 20,800 µL of 0.1N HCl in methanol containing 50 g/L of phloroglucinol and 10 g/L of ascorbic acid was added to 10 mg of sample. Samples were vortexed and incubated in a water bath at 50 °C for 20 min. The reaction was stopped by adding 1 mL of 40 mM sodium acetate in water. For the PAC polymers fraction, the volumes of reagents were multiplied by 10. Samples were filtered using a 0.22 µm PVDF filter (Chromatographic Specialities Inc., Brockville, ON, Canada).

Two microliters were injected onto an ACQUITY UPLC^®^ HSS T3 column (2.1 × 100 mm, 1.8 μm) (Waters, Milford, MA, USA) with an ACQUITY UPLC^®^ HSS T3 VanGuard pre-column (2.1 × 5 mm, 1.8 μm) (Waters, Milford, MA, USA) heated to 30 °C. The mobile phases were water (A) and acetonitrile (B), both acidified with 0.1% formic acid. The gradient was as follows, with a flow rate of 0.4 mL/min: 0–1 min: 3% B, 1–2 min: 3–4.5% B, 2–8.28 min: 4.5–16% B, 8.28–14.85 min: 16–50% B, 14.85–14.9 min: 50–95% B, 14.9–16.9 min: 95% B, 16.9–17 min: 95–3% B and 17–20 min: 3% B. UV signals were acquired at 280 nm with a resolution of 2.4 nm and a sampling rate of 20 Hz. Samples were kept at 4 °C in the autosampler compartment and injected on the same system as detailed the previous section with the same source parameters. MS data were acquired by MSE in negative electrospray ionization with a scan time of 0.2 s. The scan range (m/z) was 50 to 2000 and a collision energy ramp of 20 to 60 V was applied in the high-energy function. Each sample was injected in triplicate. Data were processed using TargetLynx XS v4.2 software.

### 2.4. Cell Culture of HepG2, Caco-2, and SH-SY5Y Cell Lines

HepG2 (passage 5 to 30) and SH-SY5Y (passage 10 to 40) cells were cultured at 37 °C/5% CO2 in Glutamax DMEM medium complemented with 10% FBS and 1X pen-strep solution. Caco-2 cells (passage 10 to 35) were cultured at 37 °C/5% CO2 in Glutamax DMEM medium complemented with 20% FBS and 1X pen-strep. ARE Reporter–HepG2 cells (passage 3 to 16) were cultured at 37 °C/5% CO2 in Glutamax DMEM medium complemented with 10% FBS, 1X pen-strep, and 0.6 mg/mL of Geneticin. Cells were grown up to 70–80% confluence then transferred in clear-bottomed 96-well microplates for 24 h at a density of 105 cells/mL (75 μL, 75,000 cells/well) for HepG2 cells, 2.6 × 105 cells/mL (75 µL, 20,000 cells/wells) for SH-SY5Y and 4 × 105 cells/mL (75 µL, 30,000 cells/wells) for Caco-2 cells.

### 2.5. Determination of Intracellular Radical Scavenging Activity Using AOP1 Bioassay

Cells were placed in presence of fresh serum-free DMEM and incubated with samples diluted for the dose–response studies in a range of concentrations (9 different concentrations obtained by serial dilutions) for 4 h at 37 °C in 5% CO_2_. An AOP1 bioassay measures the ability of an antioxidant compound to scavenge the free radicals generated intracellularly in a controlled and monitored manner (patented technology), and the effect is measured by a delay in the kinetic evolution of fluorescence emission [34]. After 4 h of incubation with samples, cells were treated for 1 h at 37 °C in 5% CO_2_ with the fluorescent biosensor. This biosensor acts as a photosensitizer, leading to moderate intracellular reactive oxygen species (ROS) production and an increase in fluorescence after light application [35].

Experiments were carried out in cell culture medium without FBS to avoid potential interaction with serum components. A least two independent experiments were performed, each on triplicate wells. The range of concentrations and the type of solvent, depending on the tested compound, are indicated in the figures. The negative controls were cells cultured without samples (control medium). For the preparation of AE and fractions A1, A2, and A3, only cell culture medium was used. For pure compound preparation, when it was used, the solvent was always inferior or equal to 1% (*v*/*v*) (ethanol 1% or DMSO 1%) in the highest tested concentration and maintained at the same proportion during the dilution process. Fluorescence (relative fluorescence unit (RFU)) at 535 nm was measured on a Varioskan Flash Spectral Scanning Multimode Reader (Thermo Fisher Scientific, Waltham, MA, USA) according to a recurrent 470 nm LED application procedure (20 iterations) of the whole 96-well plate. Raw-data kinetic profiles were recorded, and the antioxidant cell index (AOP index) was calculated from normalized kinetic profiles according to the formula (control = cell culture medium):

AOP index (%) = 100 − 100 (_0_∫^20^ RFU_sample_/_0_∫^20^ RFU_control_)

The EC_50_ was calculated with Prism8 software (GraphPad, San Diego, CA, USA) applying a non-linear regression model: log[Agonist] versus response—variable slope, according to the formula:

AOP index = AOP index_min_ + (AOP index_max_ − AOP index_min_)/(1 + 10^(Log(EC^_50_^− SC)*HS)^)

where SC is the sample concentration and HS is the Hill slope or tangent slope at the inflexion point. The best fit value of the EC_50_ was computed with 95% confidence intervals (CIs) using the asymmetrical (likelihood) method), as recommended by Prism.

### 2.6. Determination of Catalase-like Activity (or H_2_O_2_ Neutralization Assay) Using the AOP CAT Bioassay

This test is based on the intracellular presence of a nucleic acid biosensor whose fluorescence increases with H_2_O_2_ (patented technology). This bioassay evaluates the ability of an antioxidant to exert a catalase-like activity (dismutation of H_2_O_2_ in O_2_ and H_2_O) and the effect is measured by a delay in the kinetic evolution of fluorescence emission. For this, samples were tested as a range of 16 different concentrations obtained by serial dilutions and were incubated in cell culture media with H_2_O_2_ (0.34%) (*v*/*v*) for 1h at 37 °C. The resulting incubation solutions were then added to cells (14.7% of final volume) which were preliminarily incubated with the biosensor for 30 min at 37 °C in 5% CO_2_. Two independent experiments were performed, each on triplicate wells. Fluorescence (RFU) was recorded on a kinetic mode for 75 min, with T = 0 as the time of biosensor addition. Bovine liver catalase was used as positive control. The CAT-like index (fluorescence index (FI)) was calculated according to the formula (control = cell culture medium):

FI CAT-like index = 100 × [(_35_∫^75^ RFU _sample_ − _35_∫^75^ RFU _control_)^/^(_35_∫^75^ RFU _sample MAX_ − _35_∫^75^ RFU _control_)]

The EC_50_ was calculated with Prism8 software (GraphPad, San Diego, CA, USA) applying a non-linear regression model: log[Agonist] versus response—variable slope, according to the formula:

FI = FI_min_ + (FI_max_ − FI_min_)/(1 + 10^(Log(EC^_50_^−SC)*HS)^)

where SC is the sample concentration and HS is the Hill slope to calculate EC_50_, EC_10_, and EC_90_.

The confidence tab was set to compute the 95% CI using the asymmetrical method as recommended by Prism.

### 2.7. Determination of Radical Scavenging Activity at the Plasma Membrane Using CAA/DCFH-DA Bioassay

The CAA test is based on the use of a diacetate group (DA) which allows for the cell uptake of a DCFH-DA probe (2′, 7′-dichlorofluorescein-diacetate) through the plasma membrane. DCFH-DA is then cleaved by cellular esterases and DCFH is trapped within the cells. Cells are treated with a radical generator AAPH (2, 2′-azobis (2-amidinopropane) dihydro-chloride) to initiate oxidation. Peroxyl radicals produced at the plasma membrane convert non-fluorescent intracellular DCFH to its oxidized product DCF, which becomes fluorescent upon excitation [36]. The CAA test measures the decrease in cellular fluorescence intensity reflecting antioxidant effect or peroxyl radical scavenging activity in the plasma membrane [36]. Here, cells were incubated with samples (9 concentrations obtained by serial dilutions) for 1h at 37 °C in 5% CO2, in the presence of DCFH-DA (30 µM), followed by three washes, and treatment with AAPH (600 µM). Quercetin was used as a positive control. Fluorescence (RFU) was read every 5 min on a kinetic mode for as long as necessary. Dose–response curves were obtained according to the formula (control = cell culture medium):

CAA Units = 100 − (_0_∫^65^ RFU _sample_/_0_∫^65^ RFU _control_) * 100

Experiments were performed in triplicate, in culture medium without FBS, at least twice.

### 2.8. Determination of the Ability to Activate the NRF2/Keap1-Mediated Are Pathway (NRF2/ARE Bioassay)

The modulation of NRF2/ARE (ARE, Antioxidant Response Element) pathway in stably transfected HepG2 cells was performed as previously described [37]. ARE-Luc-HepG2 cells were incubated for 17 h at 37 °C in 5% CO_2_, with samples diluted in a range of 9 concentrations obtained by serial dilutions, and then treated with a mix comprising cell lysis solution and luciferin (substrate of luciferase) (BPS Bioscience, San Diego, CA, USA). Luminescence was read on a Varioskan Flash Spectral Scanning Multimode Reader. Relative luminescence unit (RLU) values reveal luciferase gene expression following the ARE promotion. Sulforaphane was used as a positive control. Results are presented as the fold increase (FI) of the negative control value (control = cell culture medium) at *t* = 20 min according to the formula:

Fold Increase FI = (RLU_sample_/RLU_control_).

The EC_50_ was calculated with Prism8 software (GraphPad, San Diego, CA, USA), applying a non-linear regression model: log[Agonist] versus response—variable slope, according to the formula:

FI = FI_min_ + (FI_max_ − FI_min_)/(1 + 10^(Log(EC^_50_^− SC)*HS)^)

where SC is the sample concentration and HS is the Hill slope, to calculate EC_50_, EC_10_ and EC_90_.

At least two independent experiments were performed, each on duplicate wells. The confidence tab was set to compute 95% CI using the asymmetrical method as recommended by Prism.

### 2.9. Statistical Analysis

At least two independent experiments, performed on triplicate wells (except for NRF2/ARE bioassay performed on duplicate wells), were performed for each cell-based assay. For the fluorescence profile figures, bars corresponding to standard deviation (SD) values were obtained from fluorescence raw data. Dose–response curves were obtained using Prism8 software (GraphPad, San Diego, CA, USA). The function used with GraphPad Prism to model the dose–response relationship is:

f(x; b,c,d,e) = c + (d − c)/(1 + 10(b(log(x) − log(EC_50_),

where the parameters c and d correspond to the lower and upper limits for mean response, respectively; the parameter e corresponds to the EC_50_, the dose leading to the half-maximal response; and parameter b determines the slope of the dose–response curve at a given dose. R^2^ represent the coefficient of determination by the non-linear regression model fit to experimental data. The asymmetrical (likelihood) method was used to compute confidence intervals (95% CIs), which is the preferred suggested choice by Prism. For the evaluation of statistical differences between EC_50_ values, we used the overlap rule for 95% CI (estimation of the statistical significance when two arms entirely overlap gives a *p* value of about 0.05 and overlap of half an arm gives a *p* value about 0.01, when *n* = 3) [38].

## 3. Results

### 3.1. Characterization of Polyphenols in Aronia Extract and Three Polyphenolic-Enriched Fractions

**Figure 1:**
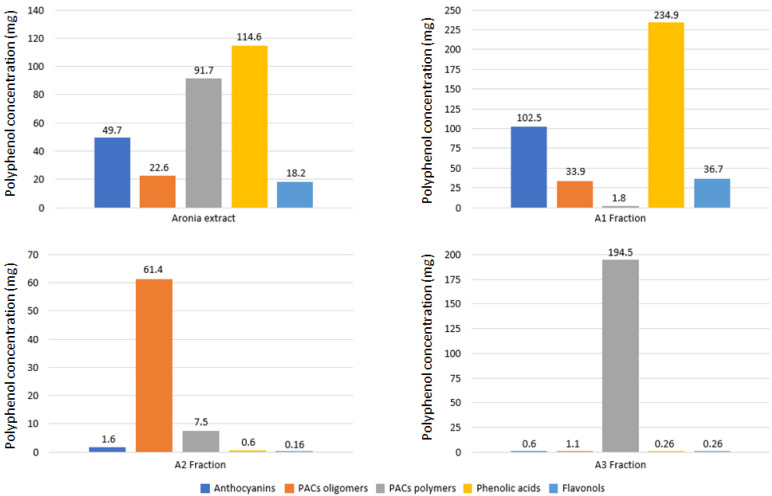
Polyphenolic characterization of AE and the three polyphenolic fractions (A1, A2, and A3). Values are expressed in milligrams of polyphenols per gram of extract or fraction.

The concentration of the main classes of polyphenols of the whole AE, the three fractions enriched in anthocyanins and phenolic acids (A1), PAC oligomers (A2), and PAC polymers (A3) is given in Figure 1. A detailed description is available in Supplementary Materials (Tables S1–S4).

The purpose of fractioning AE was to determine which polyphenols are driving the cellular antioxidative effect and whether polyphenols regulate redox status through different and/or complementary mechanisms. AE extract mainly contains PACs (21.95 g/100 g), phenolic acids (11.46 g/100 g, mainly chlorogenic acid), anthocyanins (4.97 g/100 g, predominantly cyanidin-3-galactoside), and flavonols (1.82 g/100 g, mainly glycosylated quercetin). The fractionation of AE produced three fractions with very specific and different polyphenols profiles. The A1 fraction was predominantly composed of phenolic acids (23.49 g/100 g), anthocyanins (10.2 g/100 g), and flavonols (3.67 g/100 g), with a small amount of PAC (1.69 g/100 g). The two PAC fractions contained almost only PACs, 15.15 g/100 g and 50.32 g/100 g, for A2 and A3 fractions, respectively. PACs are a complex family of polyphenols with a different degree of polymerization (DP) from monomers to complex polymers. The DP is critical for the absorption of PACs and their biological activity. The mean DP of PACs strongly differed among the four products: AE: DP = 29; A1: DP = 3; A2: DP = 11; A3: DP = 79 (Table S3).

Taken together, the three fractions obtained from the fractionation of AE showed a very different polyphenolic composition. The A1 fraction was mainly composed of phenolic acids and anthocyanins with some flavonols, whereas the two PAC fractions were almost pure PAC fractions with oligomers and polymers for A2 and A3 fractions, respectively.

### 3.2. Antioxidant Activity of the Aronia Extract and the Polyphenolic-Enriched Fractions

#### 3.2.1. Comparison of Intracellular Antioxidant Activity (Free Radical Scavenging Activity) of the Whole Aronia Extract versus Its Phenolic Fractions Assessed with AOP1 Bioassay

**Figure 2:**
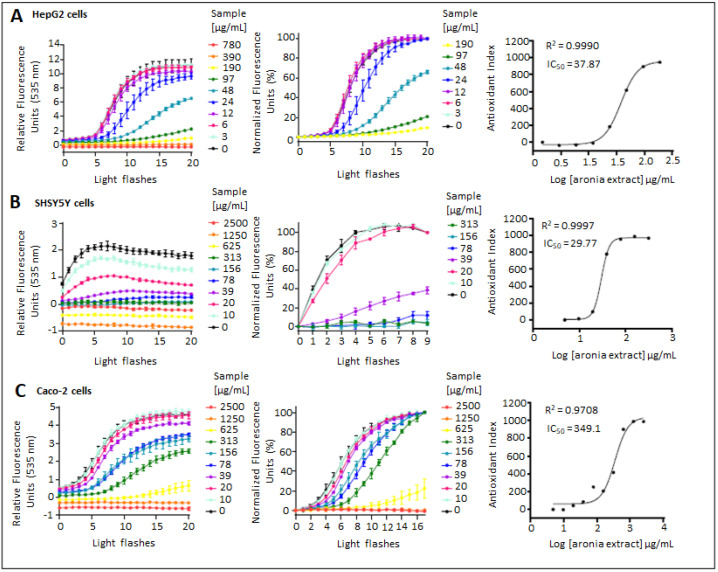
Dose–response curves of AE assessed with the AOP1 assay on different cell lines. HepG2 (panel **A**), SH-SY5Y (panel **B**) and Caco-2 cells (panel **C**) were treated for 4 h with AE in dose–response mode. Left column: kinetic profile of raw data. The raw data (RFUs, relative fluorescence units) were plotted in a kinetics-like mode showing the effect of light flashes on the fluorescence level. Data points, mean RFUs of triplicates; bars, SD. Central column: same data after normalization. Antioxidant indices correspond to control AUC minus data AUCs. Right column: antioxidant-index-based dose–response and sigmoid fit curve used for log EC_50_ estimations (µg/mL). Data points correspond to measured antioxidant indexes. EC_50_, efficacy concentration which leads to a half-maximal response; R^2^, coefficient of determination obtained by non-linear regression model fit.

Different cell lines (liver cells (HepG2), intestinal cells (Caco-2), and neuron-like cells (SH-SY5Y)) were used to assess and compare the antioxidant effect of the AE across a short panel of physiologically relevant cell models using the cell-based test called AOP1. This is a universal assay by which the antioxidant effect of extracts can be assessed accurately and quantitatively by determining the intracellular radical scavenging activity [34,35,39,40]. As shown in Table 1 and Figure 2, AE exerted antioxidant activities in the three cell lines, but the magnitude of effect varied between cell lines. AE exerted cell-specific antioxidant effects with higher effect observed in SH-SY5Y cells (EC_50_ = 29.8 µg/mL), followed by HepG2 cells (EC_50_ = 37.9 µg/mL). The lowest antioxidant effect was measured in Caco-2 cells with a value tenfold higher (EC_50_ = 349.1 µg/mL) than in the other cell lines.

The three polyphenolic fractions of AE were able to exert intracellular free radical scavenging activities in the three cell models (Table 1). The two oligo- and polymeric PAC-containing fractions (A2 and A3) exhibited the strongest antioxidant effect (lowest EC_50_ values) compared with the A1 fraction containing high amounts of anthocyanins and phenolic acids, which were almost absent from A2 and A3 fractions, whatever the cell model used. When compared with both AE and the A1 fraction, the PAC oligomer A2 fraction had a higher antioxidant effect in the three tested cell lines. The PAC polymer A3 fraction was also more effective in HepG2 and Caco-2 compared with AE as well as in HepG2 and SH-SY5Y cells when compared with A1 fraction. The antioxidant effect of A3 fraction was thus superior to the A1 fraction in Caco-2 cells.

The A1 fraction showed a heterogenous effect on the three cell types when compared with parent AE. EC_50_ values evoked a higher antioxidant effect of A1 fraction in Caco-2 cells and a lower antioxidant effect in SH-SY5Y cells, although there was no statistical difference in HepG2 cells. The two PAC fractions differed in their degree of polymerization and in the total quantity of PACs; nonetheless, the AOP1 assay did not show a statistical difference in the EC_50_ values between the oligomeric and polymeric fractions in the three cell lines (Table 1).

These results suggest that cell-based assays represent useful tools to screen for the biological antioxidant activity of complex extracts. AE exerted a free radical scavenging effect in three different cell lines. Based on the results obtained with the fractions enriched in PACs (A2 and A3), PAC oligomers and polymers play a significant role in the antioxidant capacity of polyphenols in the AE extract. However, the PACs in combination with other polyphenols in either the whole AE or the A1 fraction rich in anthocyanins and phenolic acids had a reduced efficacy. This result could be a “whole mixture effect”, with possible antagonistic interactions among polyphenols in AE [41].

#### 3.2.2. Comparison of Antioxidant Activities of Aronia Extract versus Its Polyphenolic Fractions Assessed with Catalase-like (AOPCAT) and CAA/DCFH-DA Bioassays

Another distinct antioxidant mechanism assessed for AE and polyphenolic fractions was a catalase-like assay in HepG2 cells. As shown in Table 2, AE exerted a catalase-like activity with an EC_50_ = 425.5 µg/mL. This catalase-like effect was noticeable because it is a rare antioxidant property usually limited to enzymes. The three polyphenolic fractions also displayed such antioxidant activity with, again, major differences among fractions. The A1 fraction had a significant lower effect (EC_50_ = 1186 µg/mL) compared with AE, the A2 fraction (EC_50_ = 215.8 µg/mL), and A3 fraction (EC_50_ = 180.7 µg/mL). A3 fraction showed the strongest effect with a significant difference versus the AE, whereas no difference could be detected between the two PAC fractions. AE and the three polyphenolic fractions showed no dose–response effect; therefore, no EC_50_ value could be determined CAA/DCFH-DA test (data not shown). These data suggest that the catalase-like effect of AE is mainly driven by the PACs, as shown by the higher activity of the two PAC fractions and the lower activity of the A1 fraction which is enriched in anthocyanins and phenolic acids.

#### 3.2.3. Comparison of the Indirect Antioxidant Activity of Aronia Extract and Polyphenolic Fractions Assessed with the NRF2/ARE Bioassay

**Figure 3:**
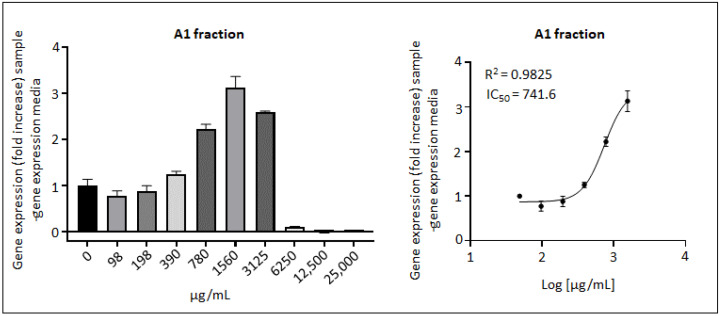
NRF2/ARE-mediated pathway activation by the fraction A1 in stable ARE-driven luciferase expression HepG2 cells. Cells were treated for 17 h with increasing doses of AE and fractions A1, A2, and A3. Only the A1 fraction showed a dose–response effect. On the left side, results are presented in gene expression fold increase compared with the control (cell culture medium only, gene expression = 1). Values under 1 indicate cytotoxicity effect. The bars are SD for mean relative luminescence units of duplicate. On the right side, the same results are presented as a dose–response curve (EC_50_: 742 µg/mL, 95% CI: [582.5, 944.9]). Data points correspond to the fold increase for each concentration and bars are SD. EC_50_ is Efficacy Concentration which leads to half-maximal response. R^2^ is the coefficient of determination obtained by non-linear regression model fit.

In addition to the assessment of direct antioxidant mechanisms (free radical or ROS scavenging activity), an indirect antioxidant mechanism was assessed through the activation of the NRF2/ARE-driven gene expression. AE did not activate the ARE sequence in stably transfected ARE-Luc HepG2 cells (EC_50_ = ND) (data not shown). In contrast to the previous direct antioxidant mechanisms (intracellular free radical scavenging and catalase-like activity), in which all the phenolic fractions exerted a measurable activity, only the A1 fraction showed an induction of the NRF2 pathway, with EC_50_ = 742 µg/mL (Figure 3). Only the A1 fraction activated the NRF2/ARE pathway, whereas the A2 and A3 fractions had no effect on this activity in this model. These data suggest that anthocyanins and phenolic acids, but not PACs, have the potential to activate this pathway.

#### 3.2.4. Antioxidant Activity of Pure Reference Compounds

##### Reference Compounds as Representative of Parent Polyphenols

The AE and the three derived polyphenolic-enriched fractions (A1, A2, and A3) showed different composition and different antioxidative effect; we thus tried to understand which polyphenols in AE could exert the antioxidant activity. The EC_50_ values of aronia fractions and pure reference compounds which could be used as markers of native polyphenols were compared. Quercetin was chosen as a reference constituent for flavonols, epicatechin was chosen for flavan-3-ols and PACs, chlorogenic acid was chosen for phenolic acids, and cyanidin 3-O-galactoside was chosen for anthocyanins.

As shown in Table 3, in terms of the scavenging capacity assessed with the AOP1 test, quercetin was clearly the strongest antioxidant among the reference compounds, in the three types of cell models (EC_50_ ranging from 1.52 µg/mL in Caco-2 and 1.79 µg/mL in SH-SY5Ycells to 6.47 µg/mL in HepG2 cells). Quercetin also exerted a catalase-like activity (EC_50_ = 57.88 µg/mL) and induced the NRF2 pathway (EC_50_ = 5.26 µg/mL). Interestingly, epicatechin had a high efficacy, particularly in Caco-2 cells (EC_50_=17.71 µg/mL), along with cyanidin-3-galactoside (EC50 = 70.38 µg/mL) and chlorogenic acid (EC_50_ = 257.5 µg/mL). Cyanidin also showed an interesting antioxidant activity in brain cells (EC_50_ = 33.02 µg/mL), which was significantly higher than the parent compound, cyanidin-3-galactoside (EC_50_ = 190.0 µg/mL). Induction of the NRF2/ARE pathway by the A1 fraction (EC_50_ = 742 µg/mL) could potentially be attributed to its enriched content in quercetin or in phenolic acids rather than its anthocyanin content (for cyanidin EC_50_ = ND). Regarding quercetin, 742 µg of the A1 fraction will bring 1.31 µg of free quercetin (2.5-fold more than in AE), which might explain the effect on NRF2 activation when considering the EC_50_ for quercetin (5.26 µg/mL).

Notably, glycosylated forms of quercetin were not tested as pure compounds, and some of these quercetin derivatives might also have an effect. Fraction A1 also contained a derivative of caffeic acid (caffeoyl quinide—1.51 µg in 742 µg of fraction A1) that might contribute to this effect.

##### Reference Compounds as Representative of Phenolic Metabolites

We finally investigated compounds corresponding to catabolites of parent polyphenols resulting from microbial metabolism in the gut. Such compounds can reach different organs, and we evaluated their antioxidant effects in intestinal, hepatic, and brain cells. Selected catabolites included 3,4-DHPVL, predominantly resulting from the breakdown of PACs as well as transferulic acid, caffeic acid, vanillic acid, hippuric acid, and cyanidin, which can be generated from phenolic acid and anthocyanin catabolism. Quercetin can also be considered a catabolite of glycosylated forms of quercetin, which are the main quercetin forms in the AE.

Caffeic and transferulic acids showed a higher scavenging activity than the parent compound, both in intestinal and brain cells (EC_50_ ranged from 142 to 173.3 µg/mL for caffeic acid) (Table 3). Caffeic acid is the only activator of the NRF2 pathway (EC_50_ = 46.01 µg/mL). Hippuric and vanillic acid did not show any effect in the different tested models. Cyanidin exhibited higher activity for radical scavenging than cyanidin-3-galactoside in intestinal cells (EC_50_ = 4.51 µg/mL and 70.38 µg/mL, respectively) and in brain cells (EC_50_ = 33.02 µg/mL and 190 µg/mL, respectively). Cyanidin did not induce the NRF2 pathway. 3,4-DHPVL exerted a free radical scavenging activity specifically in intestinal cells (EC_50_ = 259.8 µg/mL), this activity being lower than epicatechin (EC_50_ = 17.71 µg/mL). 3,4-DHPVL, similarly to epicatechin, had no activity in neuronal cells. Interestingly, 3,4-DHPVL induced the NRF2 pathway (EC_50_ = 74.55 µg/mL), whereas epicatechin had no effect. An exploratory test of 3,4-DHPVL on catalase-like activity was performed, showing some activity of this compound (EC_50_ = 320.7 µg/mL). Quercetin, as previously described, exerted strong activity in all the tested models. This compound was the most potent antioxidant compound in scavenging activity in intestinal and brain cells (EC_50_ = 1.52 µg/mL and 1.79 µg/mL, respectively), in inducing the NRF2 pathway (EC_50_ = 5.26 µg/mL) and for catalase-like activity (EC_50_ = 57.88 µg/mL).

## 4. Discussion

Epidemiological and randomized controlled intervention trials have highlighted the potential of polyphenols in aronia such as anthocyanins, PACs, and phenolic acids to prevent or attenuate chronic diseases associated with oxidative stress such as cardiovascular diseases, insulin resistance, and neurological conditions [7,14,15,16,17,42]. The in vivo relevance of an antioxidant effect has been put into question, in part due to the acellular environment widely employed to characterize this function. In living organisms, antioxidant effects can be triggered directly, by quenching free radicals, or indirectly, by activating cellular enzymatic antioxidant systems via the induction or repression of transcription factors or a combination of both, something that cannot be captured with traditional antioxidant in vitro assays such as DPPH, FRAP, or ORAC [40].

Dietary polyphenols can exert direct and indirect antioxidant effects in the gut. Dietary polyphenols such as anthocyanins, PACs, and phenolic acids are metabolized by the gut microbiota, generating catabolites with potential effects on the cellular redox environment not only in the gut, but also in distant organs (i.e., the liver and brain) following intestinal absorption. In the present study, using a combination of cellular in vitro assays, we have shown that polyphenols in AE have the potential to attenuate oxidative stress through the direct inactivation of ROS, an effect that seems to be predominantly operated by PACs rather than anthocyanins. Furthermore, indirect antioxidant effects through the activation of the NRF2 pathway were observed for the fraction rich in anthocyanins and phenolic acids and for some microbiome-generated catabolites including quercetin, caffeic acid, and 3,4-DHPVL. This is of particular relevance because the discovery of new agents that induce the NRF2 pathway are the focus of biopharmaceutical companies for treating chronic diseases [43].

AOP1 is a newly developed cellular antioxidant assay [34] based on LUCS technology which measures the ability of conditions to maintain cell homeostasis after the controlled production of oxidative stress [35]. The AOP1 bioassay uses thiazole orange acting as photosensitizer producing singlet oxygen (^1^O_2_) and hydroxyl radicals (•OH), leading to fluorescence signals proportional to the actual concentration of ROS produced by the cells [35].

Using the AOP1 assay, we were able to show that polyphenols in aronia neutralize ^1^O_2_ and •OH production in a dose-dependent manner, in intestinal, liver, and neuronal immortalized cell models. Furthermore, an interesting aspect of the results is that polyphenols in aronia displayed a catalase-like effect in HepG2 cells by inactivating H_2_O_2_ in cellular compartments and/or the culture medium. Notably, the two mechanisms displayed by aronia polyphenols may be highly relevant in vivo to protect against the highly reactive •OH radical. Indeed, H_2_O_2_ is a precursor of the •OH radical either through the reaction with certain transition metal ions (Fenton reaction) or via interaction with superoxide (Haber–Weiss reaction) [44]. Due to its high electrophilicity and high reactivity, the •OH free radical plays an important role in oxidative damage to DNA, proteins, and lipids; therefore, it is thought to play an important role in the pathophysiology of cancer, atherosclerosis, and neurodegeneration [44].

H_2_O_2_ is also a major ROS, known to be a by-product of many oxidative reactions occurring in cellular compartments such as the endoplasmic reticulum, whereas other sources of H_2_O_2_ production are the mitochondrial respiratory chain and NADPH oxidases. At a very low concentration, H_2_O_2_ can oxidase proteins (by the reversible oxidation of cystine residues), but it can also act in signaling pathways as a second messenger [45]. Thus, aronia polyphenols could protect against oxidative damage and regulate the cellular redox environment via complementary pathways by either reducing the cellular production of H_2_O_2_ or acting directly as ROS scavengers.

AE exerts a higher scavenging activity as measured by AOP1 assay in both hepatic and neuronal cells versus intestinal Caco-2 cells. Intestinal cells could be less sensitive or more resistant to the protective effect of AE against the controlled oxidative stress generated within the cells by AOP1. However, the observed effect in Caco-2 cells is of particular interest because this cell model reflects a specific interaction between all polyphenolic compounds of AE and intestinal cells before undergoing mammalian and bacterial catabolism. This protective effect could help fight against intense oxidative stress in the gastrointestinal tract in the conditions of dysbiosis induced by a poor diet, resulting in higher O_2_ concentrations [46].

To understand the relationship between different polyphenol structures and the observed antioxidant effects, we separated aronia polyphenols into different fractions. PAC-enriched fractions showed a higher potency to inactive ROS in the AOP1 and AOPCAT assay than the fraction enriched in phenolic acids and anthocyanins, indicating that PACs are largely responsible for the antioxidant effects observed in cellular models. However, great care is required when extrapolating these data to potential antioxidant effects in the different cell models tested.

PACs with DP >2 are poorly absorbed in their intact form in humans; therefore, it is unlikely that PACs in aronia, as with any other dietary source of PACs, exert direct antioxidant effects in the liver or brain cells. In contrast, PACs can reach high concentrations (≥mM) in the gastrointestinal tract, where their antioxidant effects become highly relevant. The intestinal environment is constantly exposed to ROS which are produced by epithelial cells (i.e., H_2_O_2_, NO•, superoxide), derived from foods (i.e., lipid peroxides, cytotoxic aldehydes, isoprostanes), or by the activation of immune cells by resident bacteria and toxins [47], which may increase intestinal permeability and contribute to gut dysbiosis and metabolic dysregulations involved in chronic metabolic diseases. A considerable number of studies in models of diet-induced obesity have shown that PACs protect against these metabolic imbalances [28,48,49,50]. Interestingly, a recent study using a non-invasive in situ method, with ROS-sensitive indocyanine green, demonstrated that a high-fat diet increases intestinal ROS in mice, an effect that was counteracted by supplementation with a PAC-rich extract [51]. The antioxidant effect of PACs may help protect the colonic epithelial and mucosa architecture and shape the redox environment in which certain microbes can thrive, which may partially explain the robust gut microbial changes often observed by PAC supplementation in rodents [28,31,48,49,50,52].

NRF2 has been shown to be a critical transcription factor that binds to the antioxidant response element (ARE) in the promoter region of more than 250 cytoprotective genes encoding for enzymes involved in different cellular functions (xenobiotic metabolism, heme metabolism, metabolism of carbohydrates and lipids, and the regulation of inflammation). It is also involved in the maintenance of cellular redox homeostasis such as regulatory subunits of enzymes responsible for the synthesis of cellular antioxidant glutathione, glutathione peroxidase (GPx), peroxiredoxin, and for thiol maintenance such as thioredoxin and sulfiredoxin [53]. Therefore, the induction of the NRF2/ARE pathway represents an indirect but very efficient way to activate cellular antioxidant mechanisms and counteract oxidative stress [54]. Our data suggest that unlike PACs, the fraction enriched in anthocyanins and phenolic acids promoted the translocation of NRF2 to the nucleus and activated the synthesis of antioxidant enzymes in HepG2 cells. Further analysis with individual compounds suggests that quercetin is a key contributor for this effect, whereas the major anthocyanin in AE, cyanidin 3-galactoside, showed no activity. In contrast to our results, cyanidin 3-galactoside induced NRF2 activation and the synthesis of SOD and GPx in macrophages in response to atmospheric particulate matter exposure, an ROS stimulator [55]. It is possible that the effect of cyanidin 3-galactoside is cell-specific, as observed for other compounds such as chlorogenic acid, caffeic acid, or epicatechin in the AOP1 test.

Our results indicate that the two main polyphenol classes in AE are phenolic acids and PACs. The AE fractions enriched in PACs exhibited the highest antioxidant effect. It is likely that the antioxidant effects of AE or aronia juice previously described in the literature [12,19,56] are partly due to their PACs and phenolic acid content; however, this cannot be properly assessed due to insufficient polyphenolic characterization in these studies. Aronia seems to exert its antioxidant effect through direct and indirect actions, offering a wide protection of the cell against different sources of oxidative stress. These effects can take place in the cells or cytosol by both the direct inhibition of ROS, as demonstrated in the current study, or by activation of antioxidant enzymes (catalase, superoxide dismutase, gluthatione peroxidase) [56,57]. These antioxidant effects likely play a critical role in the anti-inflammatory activity of aronia demonstrated in different animal models [19,57,58,59].

The most abundant polyphenols in aronia, namely, PACs, anthocyanins, and phenolic acids, exhibit low intestinal absorption. Instead, most of these polyphenols reach the colon and serve as substrates for the gut microbiota, which produces a complex set of catabolites which might differ between individuals according to the composition of their gut microbiota and the presence of specific bacteria harboring the enzymes responsible of the different catabolic activities [60]. We observed that cyanidin and quercetin, which can be produced by the action of bacterial glucosidases, and ferulic and caffeic acid, generated by bacterial esterases, can exert direct radical scavenging activity in Caco-2 cells. This effect was also evident for 3,4-DHPVL, a major PAC-derived metabolite. In addition, quercetin and 3,4-DHPVL showed catalase-like activity, whereas quercetin, caffeic acid, and 3,4-DHPVL were able to promote the NRF2/ARE pathway.

These results demonstrate the great versatility and different pathways that polyphenol-derived catabolites can target to modulate cellular redox homeostasis. Importantly, these catabolites are formed locally in the gastrointestinal tract; therefore, they could influence intestinal physiology before absorption [27] through antioxidant effects. Indeed, the effect of NRF2 pathway observed in liver cells could be extrapolated to intestinal tissue. Both quercetin and caffeic acid have been shown to promote NRF2 translocation in differentiated Caco-2 cells exposed to indomethacin or IFN-γ concomitant with a reduction in ROS [61,62]. In the latter study, Caco-2 cells were treated with chlorogenic acid rather than caffeic acid; however, chlorogenic acid is hydrolyzed by brush border enzymes in differentiated Caco-2 cells [63], and thus the effect on NRF2 is likely to have been mediated by caffeic acid rather than the intact molecule. Activation of the NRF2 pathway was shown to be critical to maintain gut barrier function and attenuate local and systemic inflammation in a mouse model of colitis [64].

It is important to highlight that although antioxidant effects for catabolites were observed in liver and brain cellular models, the physiological relevance of this findings needs to be taken with caution given the high EC_50_ values obtained (high µM range); the blood concentration of these metabolites through the dietary intake of aronia is likely to be in the high nM to low µM range. However, polyphenol metabolites could accumulate in different extra-intestinal tissues. For instance, the catabolite 3,4-DHPVL has been shown to cross the blood–brain barrier and accumulate in the brain tissue of rats and pigs [65]. In a mouse model of Alzheimer’s disease, 3,4-DHPVL detoxified amyloid-β oligomers and prevented memory impairment via a reduction in neuroinflammation. Although the exact mechanism behind reduced brain inflammation was not identified, given the intricate relationship between oxidative stress and neuroinflammation [66], it is reasonable to suggest that an antioxidant effect was partially involved.

Our results highlight the importance of conducting a precise characterization of the polyphenolic content in complex polyphenolic preparations such as AE to better understand the chemical composition and differential biological effects exerted by different polyphenol classes. This information is of high relevance when interpreting the effect in a human setting, because knowing the composition of polyphenols in the tested extract is critical to appreciate the degree of metabolism by the gut microbiota, identify circulating metabolites, mechanisms of action at play, and differences in the response among individuals (e.g., metabotypes). Ultimately, this approach should facilitate the development of advanced nutrition approaches to deliver nutritional bioactives such as AE or specific polyphenols (e.g., PACs) in a calibrated manner, which will help bridge the gap created by the natural heterogeneity of the polyphenol preparation and/or the gut microbiome to deliver health benefits.

Taken together, our results indicate that an AE rich in polyphenols possesses broad cellular antioxidant effects through the direct inactivation of ROS (e.g., AOP1 activity) or H_2_O_2_ (e.g., catalase-like activity) and indirect mechanisms (e.g., activation of NRF2 pathway). PACs seem to be the major contributors compared with anthocyanins and phenolic acids regarding direct antioxidant effects, whereas the fraction rich in phenolic acids and anthocyanins mediated the activation of the NRF2 pathway. Furthermore, the gut microbiome plays an important role in modulating the antioxidant effect of aronia polyphenols, because catabolites formed in the intestine may modulate the cellular redox environment in the gut and in distant organs through complementary mechanism; both direct and indirect effects were recorded for some of these catabolites (e.g., caffeic acid and quercetin).

## Figures and Tables

**Table 1:** EC_50_ values for aronia extract and its phenolic fractions assessed with AOP1 bioassay on three cell models. EC_50_ values are expressed in micrograms of product per milliliter. The 95% CIs represent asymmetrical confidence intervals, and R^2^ indicates the determination coefficient of the sigmoid regression model fit. There are no statistical differences among EC_50_ values which are marked with the same symbol. All other EC_50_ values are statistically different (*p* < 0.05).

Cell-Based Assay	AOP1								
Cell Model	HepG2 Cells			Caco-2 Cells			SH-SY5Y Cells		
EC_50_	EC_50_ (µg/mL)	R^2^	95% CI	EC_50_ (µg/mL)	R^2^	95% CI	EC_50_ (µg/mL)	R^2^	95% CI
AE	37.9	0.999	(34.9, 41.15)	349.1	0.971	(214, 1060)	29.8	0.999	(28.2, 31.51)
A1 Fraction	55.46	0.987	(34.8, 166.1)	85.6	0.989	(68.58, 112.9)	165.8	0.990	(142.8, 194.5)
A2 Fraction	11.88	0.998	(10.98, 12.81)	23.08	0.976	(16.50, 52.50)	25.57	0.998	(23.45, 27.92)
A3 Fraction	12.36	0.973	(6.24, 25.52)	38.14	0.972	(21.26, 108.7)	28.6	0.985	(19.32, 45.22)

**Table 2:** EC_50_ values for the catalase-like activities of aronia extract and its phenolic fractions assessed with AOPCAT in HepG2 cells. EC_50_ values are expressed in micrograms of product per milliliter. The 95% CI represents asymmetrical 95% confidence intervals, and R^2^ represents the determination coefficient of the sigmoid regression model fit. There are no statistical differences among EC_50_ values marked with the same symbol. All other EC_50_ values are statistically different (*p* < 0.05).

Cell-Based Assay	AOPCAT		
Cell Model	HepG2 Cells		
EC_50_	EC_50_ (µg/mL)	R^2^	95% CI
Aronia extract (AE)	425.5	0.989	(297.4, 640.2)
A1 Fraction	1186	0.980	(745.5, 12,660)
A2 Fraction	215.8	0.990	(124.6, 425.9)
A3 Fraction	180.7	0.982	(121.3, 273.6)

**Table 3:** EC_50_ values for pure individual polyphenolic compounds and metabolites assessed by AOP1 and NRF2 bioassays in different cell lines. EC_50_ values are expressed in micrograms of product per milliliter. Asymmetrical 95% CIs are indicated. R^2^ is the coefficient of determination obtained by non-linear regression model fit. ND stands for no dose–response effect and in this situation EC_50_ value could not be determined (no fit). There are no statistical differences among EC_50_ values marked with the same symbol. All other EC_50_ values are statistically different (*p* < 0.05). AOP1 in HepG2 was only assessed for quercetin (EC_50_: 6.47 µg/mL, 95% CI [6.13, 6.83] and epicatechin (EC_50_: 222.0 µg/mL, 95% CI [201.5, 244.6]. The catalase-like assay in HepG2 was only performed for quercetin (EC_50_: 57.88 µg/mL, 95% CI [ND, 80.06] and 3,4-DHPVL (EC_50_: 320.7 µg/mL, 95% CI [223.9, 430.7].

Cell-Based Assay	AOP1						NRF2/ARE		
Cell Model	Caco-2 Cells			SH-SY5Y Cells			HepG2 Cells		
EC_50_	EC_50_ (µg/mL)	R^2^	95% CI	EC_50_ (µg/mL)	R^2^	95% CI	EC_50_ (µg/mL)	R^2^	95% CI
Epicatechin	17.71	0.993	(ND, 22.82)	ND	ND	ND	ND	ND	ND
Quercetin	1.52	0.999	(1.48, 1.56)	1.38	0.998	(1.21, 1.68)	5.26	0.972	(4.37, 6.33)
Chlorogenic acid	257.5	0.970	(143.5, 926.6)	ND	ND	ND	ND	ND	ND
Caffeic acid	142.0	0.991	(114.2, 189.8)	173.3	0.991	(142.4, 222.9)	46.01	0.976	(39.72, 52.74)
Transferulic acid	189.1	0.993	(146.6, 269.3)	322.9	0.989	(261.0, 422.2)	ND	ND	ND
Hippuric acid	ND	ND	ND	ND	ND	ND	ND	ND	ND
Vanillic acid	ND	ND	ND	ND	ND	ND	ND	ND	ND
Cyanidin-3-galactoside	70.38	0.989	(49.07, 155.9)	190.0	0.980	(138.7, 261.9)	ND	ND	ND
Cyanidin	4.51	0.998	(4.05, 5.02)	33.02	0.997	(29.86, 36.52)	ND	ND	ND
3,4-DHPVL	259.8	0.992	(212, 340.9)	ND	ND	ND	74.55	0.937	(47.12, 177.4)

## Data Availability

The data presented in this study are available in the article and Supplementary Materials.

## Supplementary Materials

The following supporting information can be downloaded at: https://www.mdpi.com/article/10.3390/antiox11081561/s1, Table S1: Polyphenol composition of aronia extract and its fractions determined by UPLC-UV-QToF. Values are expressed in mg/g of extract or fraction + SD; Table S2: Identification of polyphenolic compounds by UPLC-UV-QToF in aronia extract and its fractions; Table S3: Composition in flavan-3-ols and PACs after phloroglucinolysis. Values are expressed in g/100 g of extract (mean ± standard deviation); Table S4: Anthocyanin composition of aronia extract and its polyphenolic fractions. Values are expressed in mg/g of extract (mean ± standard deviation).
