# Supplementary material for: Cellular Antioxidant Effect of an Aronia Extract and Its Polyphenolic Fractions Enriched in Proanthocyanidins, Phenolic Acids, and Anthocyanins
Source: Antioxidants (Basel). 2022 Aug 12;11(8):1561. doi: 10.3390/antiox11081561 (PMC9405024; doi:10.3390/antiox11081561)
Supplement: Supplementary file 1 [file antioxidants-11-01561-s001.zip › Supplementary tables.pdf]

Cellular Antioxidant Effect of an Aronia Extract and Its Polyphenolic Fractions Enriched in Proanthocyanidins, Phenolic Acids, and Anthocyanins

Cécile Dufour [1], Jose A. Villa-Rodriguez [2,*], Christophe Furger [1], Jacob Lessard-Lord [3], Camille Gironde [1], Mylène Rigal [1], Ashraf Badr [3], Yves Desjardins [3] and Denis Guyonnet [2]

[1] Anti Oxidant Power AOP/MH2F-LAAS/CNRS, 7 Avenue du Colonel Roche, BP 54200, 31031 Toulouse, France
[2] Business Incubation Group, Symrise Taste, Nutrition & Health, 1E Allée Ermengarde d'Anjou, 35000 Rennes, France
[3] Institute of Nutrition and Functional Foods (INAF), Université Laval, Quebec, QC G1V 0A6, Canada
* Correspondence: jose.villa.rodriguez@symrise.com; Tel.: +33-02-56-01-64-60

Table S1. Polyphenol composition of aronia extract and its fractions determined by UPLC-UV-QToF. Values are expressed in mg/g of extract or fraction ± standard deviation.

	**Concentration (mg/g) (Mean ± Standard Deviation)**			
	**Aronia Extract**	**A1 Fraction**	**A2 Fraction**	**A3 Fraction**
Catechin	0.140 ± 0.004	0.325 ± 0.011	0.736 ± 0.008	ND
Epicatechin	1.25 ± 0.04	2.10 ± 0.03	4.31 ± 0.10	0.81 ± 0.02
Flavan-3-ols monomers total	1.39 ± 0.04	2.42 ± 0.04	5.05 ± 0.10	0.81 ± 0.02
Procyanidin B2	2.213 ± 0.010	3.80 ± 0.02	1.50 ± 0.04	0.111 ± 0.010
Neochlorogenic acid	53.2 ± 0.2	108.4 ± 0.4	0.221 ± 0.006	0.138 ± 0.001
Chlorogenic acid	53.7 ± 0.2	111.2 ± 0.4	0.154 ± 0.004	0.125 ± 0.001
Cryptochlorogenic acid	4.624 ± 0.014	9.20 ± 0.05	ND	ND
Caffeoyl quinide	1.122 ± 0.013	2.03 ± 0.03	0.205 ± 0.002	ND
Coumaroyl quinic acid	1.582 ± 0.005	3.116 ± 0.015	ND	ND
Vanilloyl hexose	0.253 ± 0.008	0.566 ± 0.013	ND	ND
3,4-dihydroxybenzoic acid	0.121 ± 0.001	0.299 ± 0.003	ND	ND
**Phenolic acids total**	**114.6 ± 0.5**	**234.9 ± 1.0**	**0.580 ± 0.011**	**0.263 ± 0.002**
Quercetin-pentoside-hexoside	2.579 ± 0.002	5.01 ± 0.03	0.049 ± 0.001	ND
Quercetin hexoside deoxyhexoside 1	2.390 ± 0.008	4.77 ± 0.02	ND	ND
Quercetin-3-galactoside	3.978 ± 0.013	8.08 ± 0.04	0.049 ± 0.001	ND
Quercetin-3-rutinoside (Rutin)	2.291 ± 0.007	4.56 ± 0.02	ND	ND
Quercetin-3-glucoside	2.964 ± 0.009	5.98 ± 0.03	ND	ND
Quercetin-dihexoside 1	1.687 ± 0.004	3.287 ± 0.013	ND	ND
Quercetin-dihexoside 2	0.698 ± 0.002	1.470 ± 0.007	ND	ND
Quercetin	0.693 ± 0.004	1.76 ± 0.04	0.066 ± 0.001	ND
Quercetin-hexoside-deoxyhexoside 2	0.210 ± 0.001	0.407 ± 0.003	ND	ND
Quercetin-pentoside	0.194 ± 0.001	0.392 ± 0.002	ND	ND
Isorhametin-hexoside-deoxyhexoside 1	0.254 ± 0.004	0.512 ± 0.007	ND	ND
Isorhametin-hexoside-deoxyhexoside 2	0.233 ± 0.003	0.491 ± 0.002	ND	ND
**Flavonols total**	**18.17 ± 0.05**	**36.7 ± 0.2**	**0.165 ± 0.002**	**ND**

Table S2. Identification of the polyphenolic compounds by UPLC-UV-QToF in aronia extract and its fractions.

Polyphenol class	Identification	RT (min)	[M-H]⁻	Molecular formula	Error (ppm)	MS2 Fragments (Relative Intensity)	Level *
Flavan-3-ols and PACs	Catechin	7.39	289.0711	C15H14O6	−0.35	203.0706 (100); 245.0814 (57)	1
	Epicatechin	10.81	289.0715	C15H14O6	1.04	245.0814 (100)	1
	Procyanidin B2	9.52	577.1348	C30H26O12	0.35	289.0717 (100); 407.0768 (87); 425.0872 (30); 245.0814 (23)	1
Phenolic acids	Neochlorogenic acid	5.06	353.0873	C16H18O9	0.73	191.0555 (100); 135.0447 (89); 179.0332 (57)	1
	Chlorogenic acid	8.08	353.0871	C16H18O9	−0.96	191.0561 (100)	1
	Cryptochlorogenic acid	8.90	353.0870	C16H18O9	−1.80	135.0451 (100); 173.0455 (67); 179.0350 (54); 191.0565 (53)	1
	Caffeoyl quinide	12.95	335.0765	C16H16O8	−1.38	161.0241 (100); 133.0290 (50); 135.0444 (18)	2
	Coumaroyl quinic acid	7.01	337.0918	C16H18O8	−1.99	163.0403 (100); 119.0502 (82); 191.0561 (16)	2
	Vanilloyl hexose	7.67	329.0904	C14H18O9	−2.1	167.0350 (100); 123.0470 (34)	2
	3,4-dihydroxybenzoic acid	3.48	153.0184	C7H6O4	−2.61	109.0280 (100)	1
Flavonols	Quercetin-pentoside-hexoside	16.26	595.1302	C26H28O16	−0.21	300.0275 (100); 271.0233 (14); 255.0286 (7)	2
	Quercetin hexoside deoxyhexoside 1	17.01	609.1432	C27H30O16	−1.89	300.0275 (100); 271.0233 (12); 255.0281 (6)	2
	Quercetin-3-galactoside	17.27	463.0883	C21H20O12	−2.53	300.0275 (100); 271.0247 (39); 255.0297 (16)	1
	Quercetin-3-rutinoside	17.51	609.1453	C27H30O16	−0.98	300.0275 (100); 271.0238 (11)	1
	Quercetin-3-glucoside	17.80	463.0893	C21H20O12	−2.24	300.0275 (100); 271.0249 (38); 255.0300 (16)	1
	Quercetin-dihexoside 1	13.88	625.1396	C27H30O17	−0.01	300.0275 (100); 271.0237 (13)	2
	Quercetin-dihexoside 2	14.26	625.1417	C27H30O17	0.14	300.0275 (100); 271.0243 (12)	2
	Quercetin	23.46	301.0356	C15H10O7	2.66	151,0050 (100)	1
	Quercetin-hexoside-deoxyhexoside 2	19.22	609.1449	C27H30O16	−1.91	315.0477 (100); 314.0428 (37); 299.0205 (37); 271.0213 (24); 285.0393 (13)	2
	Quercetin-pentoside	18.83	433.0763	C20H18O11	−1.85	300.0275 (100); 271.0239 (55); 255.0292 (23)	2
	Isorhametin-hexoside-deoxyhexoside 1	19.83	623.1608	C28H32O16	−1.55	315.0511 (100); 314.0417 (55)	2
	Isorhametin-hexoside-deoxyhexoside 2	20.10	623.1615	C28H32O16	−0.30	315.0486 (100); 314.0384 (33)	2

* Identification levels were established according to Sumner et al. Level 1 identifications were validated by an authentic standard, while level 2 identifications were proposed according to exact mass, MS/MS fragmentation and UV absorption.

Table S3. Composition in flavan-3-ols and PACs after phloroglucinolysis. Values are expressed in g/100 g of extract (mean ± standard deviation).

	Concentration (g /100 g)			
	**Aronia Extract (AE)**	**A1 Fraction**	**A2 Fraction**	**A3 Fraction**
Catechin	0.014 ± 0.001	0.033 ± 0.001	0.074 ± 0.001	ND
Epicatechin	0.125 ± 0.004	0.210 ± 0.003	0.431 ± 0.010	0.081 ± 0.002
Flavan-3-ols monomers total	0.139 ± 0.004	0.242 ± 0.004	0.505 ± 0.010	0.081 ± 0.002
Procyanidin B2	0.221 ± 0.001	0.380 ± 0.002	0.150 ± 0.004	0.011 ± 0.001
PACs total	21.95 ± 0.02	1.69 ± 0.10	15.15 ± 0.04	50.32 ± 0.05
Mean DP	29 ± 0.6	3 ± 0.6	11 ± 0.7	74 ± 6

Table S4. Anthocyanin composition of aronia extract and its polyphenolic fractions. Values are expressed in mg/g of extract (mean ± standard deviation).

	Concentration (mg /g)			
	**Aronia Extract (AE)**	**A1 Fraction**	**A2 Fraction**	**A3 Fraction**
Cyanidin-3-galactoside	31.8 ± 0.9	64 ± 3.9	0.89 ± 0.03	0.33 ± 0.002
Cyanidin-3-glucoside	2.44 ± 0.05	5.0 ± 0.3	0.168 ± 0.011	0.022 ± 0.001
Cyanidin-3-arabinoside	13.2 ± 0.3	28.6 ± 1.8	0.423 ± 0.006	0.171 ± 0.003
Cyanidin-3-xyloside	2.21 ± 0.06	4.9 ± 0.3	0.081 ± 0.001	0.029 ± 0.001
Peonidin-3-galactoside	ND	ND	ND	ND
Peonidin-3-glucoside	ND	ND	ND	ND
Peonidin-3-arabinoside	ND	ND	ND	ND
Pelagornidin-3-glucoside	ND	ND	ND	ND
Pelagornidin-3-rutinoside	ND	ND	ND	ND
**Total Anthocyanins**	**49.7 ± 1.3**	**102 ± 6**	**1.56 ± 0.05**	**0.552 ± 0.007**
